# Design and Characterization of Aromatic Copolyesters Containing Furan and Isophthalic Rings with Suitable Properties for Vascular Tissue Engineering

**DOI:** 10.3390/ijms26136470

**Published:** 2025-07-04

**Authors:** Edoardo Bondi, Elisa Restivo, Michelina Soccio, Giulia Guidotti, Nora Bloise, Ilenia Motta, Massimo Gazzano, Marco Ruggeri, Lorenzo Fassina, Livia Visai, Gianandrea Pasquinelli, Nadia Lotti

**Affiliations:** 1 Department of Civil, Chemical, Environmental, and Materials Engineering, University of Bologna, Via Terracini 28, 40131 Bologna, Italy; edoardo.bondi3@unibo.it (E.B.); m.soccio@unibo.it (M.S.); nadia.lotti@unibo.it (N.L.); 2 Molecular Medicine Department (DMM), Centre for Health Technologies (CHT), Unità di Ricerca (UdR) INSTM, Operative Unit (OU) of Interuniversity Center for the Promotion of the 3Rs Principles in Teaching and Research (Centro 3R), University of Pavia, 27100 Pavia, Italy; elisa.restivo01@universitadipavia.it (E.R.); livia.visai@unipv.it (L.V.); 3 UOR6 Nanotechnology Laboratory, Department of Prevention and Rehabilitation in Occupational Medicine and Specialty Medicine, Istituti Clinici Scientifici Maugeri IRCCS, 27100 Pavia, Italy; 4 Department of Medical and Surgical Sciences (DIMEC), University of Bologna, Via Massarenti 9, 40138 Bologna, Italy; ilenia.motta2@unibo.it (I.M.); gianandr.pasquinelli@unibo.it (G.P.); 5 Institute for Organic Synthesis and Photoreactivity, ISOF-CNR, Via Gobetti 101, 40129 Bologna, Italy; massimo.gazzano@isof.cnr.it; 6 Department of Drug Sciences, University of Pavia, Viale Taramelli 12, 27100 Pavia, Italy; marco.ruggeri@unipv.it; 7 Department of Electrical, Computer and Biomedical Engineering, University of Pavia, Via Ferrata 5, 27100 Pavia, Italy; lorenzo.fassina@unipv.it; 8 Pathology Unit, IRCCS Azienda Ospedaliero-Universitaria di Bologna, 40138 Bologna, Italy

**Keywords:** poly(butylene 2,5-furanoate), poly(butylene isophthalate), aromatic copolyesters, hydrolytic stability, biocompatibility, hemocompatibility

## Abstract

Cardiovascular diseases are responsible for a large number of severe disability cases and deaths worldwide. Strong research in this field has been extensively carried out, in particular for the associated complications, such as the occlusion of small-diameter (<6 mm) vessels. Accordingly, in the present research, two random copolyesters of poly(butylene 2,5-furandicarboxylate) (PBF) and poly(butylene isophthalate) (PBI), were successfully synthesized via two-step melt polycondensation and were thoroughly characterized from molecular, thermal, and mechanical perspectives. The copolymeric films displayed a peculiar thermal behavior, being easily processable in the form of films, although amorphous, with T_g_ close to room temperature. Their thermal stability was high in all cases, and from the mechanical point of view, the materials exhibited a high ultimate strength, together with values of elastic moduli tunable with the chemical composition. The long-term stability of these materials under physiological conditions was also demonstrated. Cytotoxicity was assessed using a direct contact assay with human umbilical vein endothelial cells (HUVECs). In addition, hemocompatibility was tested by evaluating the adhesion of blood components (such as the adsorption of human platelets and fibrinogen). As a result, a proper chemical design and, in turn, both the solid-state and functional properties, are pivotal in regulating cell behavior and opening new frontiers in the tissue engineering of soft tissues, including vascular tissues.

## 1. Introduction

Cardiovascular diseases (CVDs) continue to be the leading cause of mortality in countries belonging to the European Society of Cardiology (ESC), resulting in over 3 million deaths in 2021, of which more than 1.6 million occurred in women and 1.5 million in men [1]. CVD-related mortality rates were disproportionately higher in middle-income ESC member countries than in high-income countries, affecting 53% of women and 46% of men in middle-income areas compared to 34% of women and 30% of men in high-income regions [1]. This persistently high CVDs incidence is primarily driven by aging populations and an ongoing trend toward urbanization, which introduces increased exposure to environmental pollutants, in particular air pollution. In 2021, healthcare and social care costs attributed to CVDs in the European Union (EU) alone reached 155 billion euros, accounting for 11% of the EU’s total healthcare expenditure [1].

Among CVDs, peripheral artery disease (PAD) affects 113 million people aged 40 and over worldwide. The overall prevalence is 1.52%, which increases with age, reaching 14.91% in individuals between 80 and 84 years [2]. To date, the most common procedure to restore proper blood flow, in particular in the case of chronic limb-threatening ischemia (CLTI), one of the most severe forms of PAD, involves revascularization with endovascular procedures. However, in cases of long lesions, calcified arteries and chronic occlusions, open surgery remains the best choice [2,3].

Conventional vascular grafts, consisting of human tissue taken directly from the patient (autologous) or from donors (homologous), may offer superior biocompatibility and low immune response. However, these grafts are not always available, especially in patients with advanced disease or in cases where healthy vessels are not suitable for harvesting [4,5].

Therefore, the development of synthetic vascular grafts is of great importance; however, in the case of small-diameter vascular grafts (d < 6 mm), some issues are still unresolved, often associated with complications like calcification, thrombosis, intimal hyperplasia, and aneurysm formation, resulting in a high failure rate [6,7,8,9].

The proper choice of the most appropriate biomaterials for producing artificial solutions is crucial. In this regard, synthetic polyesters offer several advantages owing to their wide range of physico–chemical properties and high versatility in terms of their peculiar applications. Moreover, most of these polyesters are biocompatible, which is mandatory for biomedical applications. Finally, the choice of aromatic polyesters instead of aliphatic ones is preferable for long-term applications, such as the realization of vascular grafts. Indeed, the presence of aromatic rings in their backbone provides high durability and stability over time.

For example, terephthalic acid-based aromatic polyesters, such as poly(ethylene terephthalate) (PET, commercially known as Dacron^®^), have been widely used in vascular applications, and in particular for large-diameter vascular grafts [10,11], even though they present some complications in small-diameter grafts, primarily due to thrombosis [12]. For these reasons, improved techniques have been explored over time, moving towards chemical and surface modification [13,14,15] and the research of new alternative aromatic polyesters.

2,5-furan dicarboxylic acid (FDCA) is a biomass-derived monomer included in the top 12 biobased monomers list issued by the US Department of Energy in 2004, and is advocated as a greener alternative to terephthalic acid [16], as it can be completely derived from renewable feedstocks, biomass, and sugars containing six carbon atoms [17]. The extensive research on this building block is supported by a rich literature, according to which a wide plethora of sustainable homo- and copolyesters has been deeply investigated. Among these materials, poly(butylene 2,5-furanoate) (PBF), the green *alter-ego* of fossil-based poly(butylene terephthalate) (PBT), is a homopolymer of particular interest owing to its good mechanical properties and high thermal stability [18,19,20,21,22,23,24,25,26].

Another interesting aromatic building block is isophthalic acid, an isomer of terephthalic acid, with carboxyl groups in positions 1,3 in the aromatic ring, instead of 1,4, as in the case of terephthalic acid. Isophthalic acid is commonly obtained from fossil resources; however, several processes based on fermentation and renewable resources have been reported [27]. This monomer is employed in the synthesis of poly(butylene isophthalate) (PBI), another interesting alternative to PBT and PET [28,29], due to its excellent mechanical behavior, high stability, and easy melt processability [30,31].

Based on their characteristics, both PBF and PBI are interesting candidates to extend the list of potential materials used to obtain small-diameter vascular grafts. However, to the best of our knowledge, very few investigations into the biocompatibility of these polyesters [32,33] have been performed, and no data on their hemocompatibility are available to date. To finely tune the properties of both homopolymers while maintaining at the same time, their already good properties, copolymerization is a successful, widely used, and reproducible technique that is easy to scale up at the industrial level.

In the present study, PBF, PBI, and their two random copolymers, poly(butylene furanoate/isophthalate), P(BF_x_BI_y_), where x and y represent the relative molar amounts of the two comonomeric units, were synthesized by two-stage melt polycondensation and then characterized from molecular, structural, thermal, and mechanical perspectives. Their surface wettability and stability to hydrolytic degradation were also investigated. Finally, to evaluate the necessary requirements of biocompatibility and hemocompatibility, in view of potential applications as vascular grafts [34,35], preliminary in vitro cytotoxicity tests were carried out, and human platelet adhesion as well as plasma fibrinogen binding were investigated to check the response of the designed materials to blood contact and if any difference between them occurred. The different chemical structures of the two comonomers, together with the different natures of the two parent homopolymers, are expected to result in different solid-state and functional properties of the final materials.

## 2. Results and Discussion

Two aromatic homopolyesters, PBF and PBI, and two random copolymers, P(BF_90_BI_10_) and P(BF_10_BI_90_), with different molar ratios between the diacid counterparts, were successfully synthesized via melt polycondensation. The final properties, from the chemico-physical to the more functional ones, were correlated to the different chemical compositions.

### 2.1. Molecular Characterization

To confirm the chemical structures of the synthesized homopolymers (PBF and PBI) and two copolymers (P(BF_90_BI_10_) and P(BF_10_BI_90_)) and to calculate their actual compositions, ^1^H-NMR analysis was performed. All spectra were consistent with the theoretical structures, proving good control over the polymerization process.

**Figure 1:**
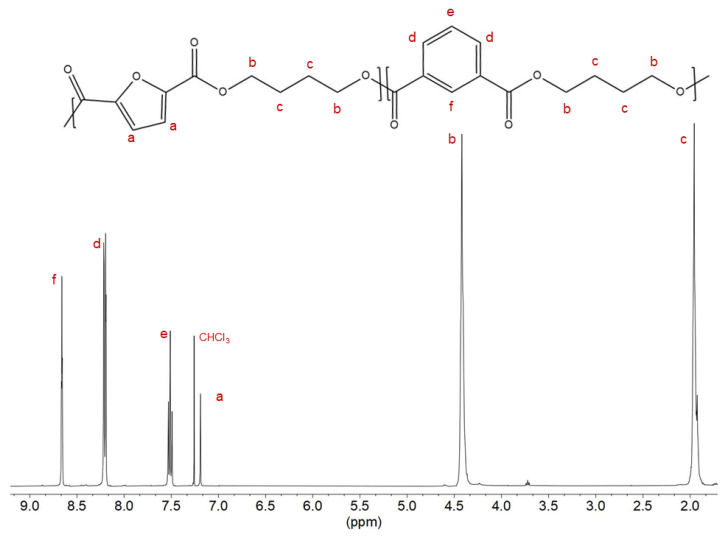
^1^H-NMR spectrum of P(BF_10_BI_90_) with peaks’ assignment.

In Figure 1, the spectrum of P(BF_10_BI_90_), together with the relative peak assignments, is shown as an example, while the spectra of the parent homopolymers are shown in Figure S1. In addition to the chloroform signal located at δ 7.25 ppm, only the characteristic peaks of each co-unit were observed in all spectra. The furan ring shows a singlet at δ 7.20 ppm (*a*, 2 H), while the isophthalic ring shows a singlet at δ 8.66 ppm (*f*, 1 H), two doublets at δ 8.20 ppm (*d*, 2 H), and a triplet at δ 7.52 ppm (*e*, 1 H). In the latter case, a typical pattern of a meta-disubstituted benzene ring was observed. For both aliphatic glycolic portions, the protons in the α-position to the ester oxygen are evident at δ 4.4 ppm (*b*, triplet, 4 H), due to the deshielding effect of the oxygen atoms, while those in the β-position are located at δ 1.9 ppm (*c*, multiplet, 4 H). For the two copolymers, the actual molar composition was obtained from the relative ratio between the normalized intensities of the *d* peak, referring to the isophthalic subunit, and the *a* peak for the furan subunit. As can be seen from the data in Table 1, the effective composition was very similar to or even the same as the feed one, confirming the good control over the synthetic process.

Viscometric analyses in dilute solution were carried out for all samples to obtain an indirect estimation of the molecular weight of the synthesized materials. The values of intrinsic viscosity (I.V., Table 1) ranged between 0.77 and 1.13, in line with high and comparable molecular weights. GPC measurements were also performed for PBI and P(BF_10_BI_90_), the only polymers soluble in chloroform. Through this analysis, high molecular weights were obtained, together with polydispersity index (PDI) values similar and below 2 (Table 1), in line with those typical of polymers obtained by polycondensation, as further confirmation of the good control over polymerization.

### 2.2. Thermal Analysis

**Figure 2:**
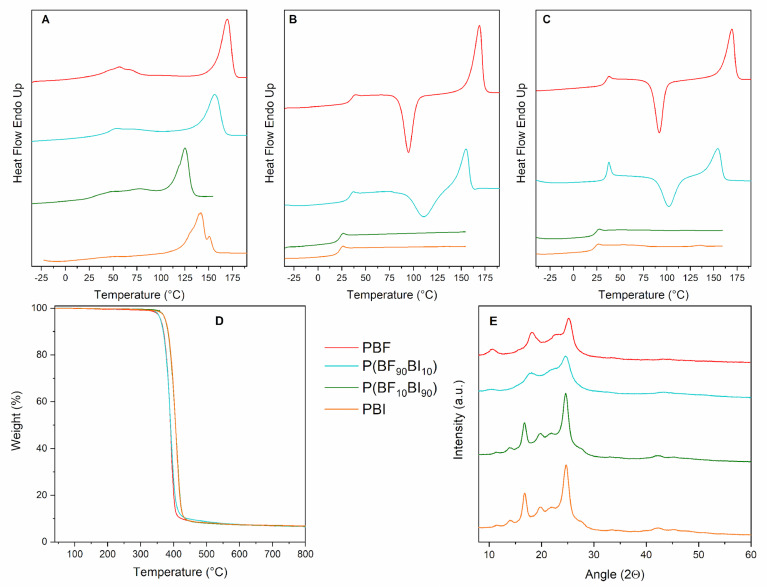
First (**A**) and second (**B**) DSC scans of purified powders, and (**C**) first DSC scan of films of PBF, PBI, and P(BF_x_BI_y_) copolymers. TGA (**D**) and WAXS (**E**) traces of PBF, PBI, and P(BF_x_BI_y_) copolymers, purified powders.

The TGA thermograms obtained from the purified powders are shown in Figure 2D, and the temperatures of the onset of degradation (T_id_) and maximum weight loss rate (T_max_) are listed in Table 2. All the materials exhibited the same one-step degradation profile, although some differences in thermal stability were observed between the two pairs of similar polymers. In fact, both PBF and P(BF_90_BI_10_) are characterized by a similar T_id_ in the range 375–378 °C and the same value of T_max_, 392 °C. PBI and P(BF_10_BI_90_) showed higher T_id_ and T_max_ values, in the range 387–389 °C and 409 °C, respectively. Therefore, copolymerization did not compromise the excellent thermal stability of the starting homopolymers for each pair.

The higher thermal stability of PBI and the copolymer rich in BI co-units compared to that of PBF and the P(BF_90_BI_10_) copolymer can be explained by the higher resonance energy of the benzene ring (E_ris,benzene_ = 36 kcal/mol) [36,37] with respect to that of the furan ring (E_ris,furan_ = 16 kcal/mol) [37]. The lower resonance energy of the heterocyclic ring is related to the presence of the highly electronegative oxygen atom, which hinders the delocalization of π-electrons. It should be noted that, in all cases, the characteristic degradation temperatures are close to those of PET [38,39], which is commonly used as a starting material for artificial vascular grafts.

Finally, all TGA curves at high temperature (800 °C) were characterized by a residue of about 10%.

The purified powders were then characterized from a calorimetric point of view to obtain information about their main thermal transitions and their capability to crystallize. The I and II scan DSC traces are shown in Figure 2A and 2B, respectively, and the associated thermal data are listed in Table 2.

As observed from the first scan, all the materials were semicrystalline (Figure 2A), as the DSC curves showed an endothermic baseline jump associated with the glass-to-rubber transition, followed by an endothermic peak at a higher temperature attributable to the melting of the crystalline portion. As far as the homopolymers are concerned, the values of T_m_ and ΔH_m_ (Table 2) of PBF are higher than those of PBI.

As is well known, inter-chain interactions [40] are one of the various factors influencing thermal properties, being responsible for strong molecular cohesion and therefore affecting the mobility of macromolecular chains. Accordingly, π-π stacking between the aromatic rings is possible for both aromatic polyesters under study [41,42,43], resulting in a highly dense-packed structure. Moreover, in PBF, the formation of intermolecular hydrogen bonds is also possible, due to the presence of the electronegative oxygen atom inside the furan ring [21,44,45]. This results in even better packing of the PBF chains and, therefore, a higher melting temperature.

The copolymers exhibited lower melting temperatures (T_m_) and melting enthalpies (ΔH_m_) than their reference homopolymers. This is not surprising, considering that the introduction of a co-unit inside a homopolymer results in the formation of less perfect crystals (lower T_m_) and hinders the ability of the macromolecular chains to crystallize (lower ΔH_m_).

In addition, a weak endothermic signal was detected between T_g_ and T_m_ for all materials except PBI. According to previous studies [46,47,48], this phenomenon could be ascribed to the isotropization of a 1D- or 2D-ordered phase, whose development is possible owing to the presence of mesogenic groups consisting of furan [21,49,50] and isophthalic [51,52] moieties, together with flexible units such as aliphatic butylene segments.

The second heating scan was performed after rapid cooling from the melt to minimize the crystallization process and better evaluate the glass transition phenomenon. According to the obtained traces (Figure 2B), all materials were amorphous, except for PBF, which retained some residual crystallinity. In addition, some differences in the crystallization capabilities of the two families of polyesters were detected. More specifically, from the II scan profiles of PBF and P(BF_90_BI_10_), a similar T_g_ of 34 °C and 33 °C, respectively, can be observed, indicating a glassy amorphous phase. Moreover, the low amount of butylene isophthalate co-unit was not sufficient to significantly alter the value of the glass transition temperature. Moreover, after the T_g_ jump, an exothermic crystallization peak was observed, followed by a melting endotherm. As mentioned before, by directly comparing the values of enthalpy associated with crystallization and melting phenomena, in the case of PBF, the enthalpy of crystallization is lower than that of melting (ΔH_m_ > ΔH_cc_) (Table 2), indicating that some crystallization occurred even during cooling. In contrast, for P(BF_90_BI_10_), the melting phenomenon only involves the crystals that formed during heating (ΔH_m_ = ΔH_cc_), confirming that, in this case, the cooling rate of 100 °C/min was sufficient to lock the chains in an amorphous state. Interestingly, the crystallization temperature was higher for the P(BF_90_BI_10_) copolymer than for PBF (110 vs. 95 °C), proving that the macromolecular chains require more energy to crystallize due to the introduction of the BI co-units, which hinder the rearrangement of the PBF chains into ordered crystalline structures. Conversely, the T_m_ values were in line with those measured in the first scan, and the differences between the two values were directly ascribed to copolymerization.

PBI and P(BF_10_BI_90_) showed a lower ability to crystallize; in fact, in both cases, the II DSC scan curves showed only the glass transition phenomenon at 22 °C and 24 °C, respectively. These values, which are close to the room temperature, indicate a partially mobile amorphous phase. The lower T_g_ values of PBI and P(BF_10_BI_90_) than those of PBF and P(BF_90_BI_10_) can again be attributed to the higher interchain interactions (π-π stacking and interchain hydrogen bonds) present in the two furan-based polyesters.

DSC measurements were also performed on the compression-molded films to investigate how the different processing methods affected the main thermal transitions of the materials under study. Figure 2C shows the I-scan DSC traces of the films, while the relative calorimetric data are reported in Table 3. All materials were amorphous. However, PBF and P(BF_90_BI_10_) crystallized during the heating scan, while the DSC traces of the other two materials only showed an endothermic jump relative to the glass-to-rubber transition, with values in line with those already observed for purified powders. In this case, the crystallization temperature was higher for P(BF_90_BI_10_) than for PBF (102 vs. 92 °C). Interestingly, despite being amorphous and rubbery, PBI and P(BF_10_BI_90_) can be easily processed into free standing films.

Finally, the second-scan behavior of all films was similar to that of the corresponding powders.

### 2.3. Wide Angle X-Ray Scattering (WAXS)

In order to analyze the nature of the crystalline phase present in the materials under investigation and determine their degree of crystallinity, X-ray diffractometric analysis (WAXS) was performed on the purified powders. The diffraction patterns are shown in Figure 2E, and the degree of crystallinity χ_c_ is reported in Table 2.

As can be seen, all the profiles are typical of semicrystalline polymers, with reflections of different width and intensity emerging from a bell-shaped profile relative to the amorphous portion of the material. In detail, PBF shows two intense reflections at 2θ 18.2° (010) and 25.2° (100), a shoulder at 22.7°, which concerns the diagonal planes, and a small peak at 10.7° (001), as demonstrated by Zhu et al. [19]. In contrast, the WAXS pattern of PBI shows several reflections, with the two most intense located at 2θ 16.8° and 24.7°. The profile of the copolymer rich in the BF co-unit shows peaks characteristic of the PBF phase, while the diagram of the sample rich in the BI co-unit is typical of the PBI crystal phase. However, in the former case, the position of the reflections shifts to lower angular values (i.e., larger lattice distances), suggesting that the presence of the BI co-unit widens the lattice planes; in parallel, the Crystal Size (C.S.) slightly decreases (Table 2), indicating that crystal growth is disturbed. Conversely, in the latter case, the peaks were in the same position as those of the two reference homopolymers, proving the total exclusion of the BF co-units from the main crystalline phase of PBI. In both cases, the reflections were slightly less intense and broader than those of the respective reference homopolymers, indicating a less perfect crystalline phase, as confirmed by the X_c_ values (Table 2). Furthermore, the developed crystalline phase was always unique, of PBF in the P(BF_90_BI_10_) copolymer, and of PBI in the P(BF_10_BI_90_) copolymer. In contrast to the case of powders, the diffractometric profiles of the films exhibited only a bell-shaped profile, which is typical of amorphous materials. The mean dimension of the crystal domains, as estimated from the peak widths, is roughly twice that of the samples belonging to the PBI crystal phase with respect to the PBF phase (Table 2). Moreover, the presence of co-units causes a reduction of 20% in the C.S. of the P(BF_90_BI_10_) copolymer compared to that of the parent homopolymer, whereas it seems to have no effect on the P(BF_10_BI_90_) sample.

### 2.4. Water Contact Angle (WCA) Measurements

The compression-molded films were subjected to water contact angle (WCA) measurements under static conditions to assess the hydrophilicity/hydrophobicity of their surfaces. The contact angle values obtained are shown in Table 3, and photos of the droplets deposited on the surface of each film are shown in Figure S2.

All materials were hydrophobic (WCA > 90°), with PBI showing the highest WCA value of 96°. The increased wettability observed for furan-containing materials can be explained by the presence, in the furan ring, of a dipole moment, as a result of the presence of a polar furan ring.

Notably, all these WCA values are between those of the most common materials used for the production of synthetic vascular grafts, such as PET (WCA values in the range 81–84°) [53,54] and the more hydrophobic PTFE (WCA values in the range 111–114°) [55,56].

### 2.5. Mechanical Characterization

**Figure 3:**
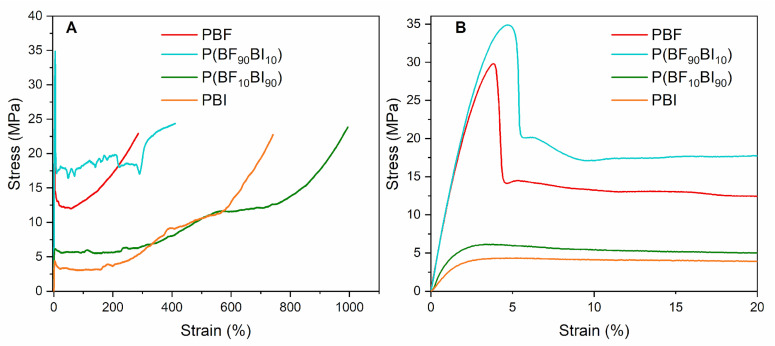
Stress–strain curves (**A**) and enlargement of the low-stress region (**B**) of PBF, PBI, and P(BF_x_BI_y_) copolymeric films.

In order to obtain information on the mechanical properties of the synthesized materials, tensile tests were conducted on PBF, PBI, and their copolymers in the form of films. The obtained stress–strain curves are shown in Figure 3, and the mechanical characterization data (elastic modulus, E, stress at break, σ_B_, strain at break, ε_B_, stress at yield, σ_Y_, and strain at yield, ε_Y_) are reported in Table 4.

For the homopolymers, PBF is characterized by a higher value of elastic modulus, similar stress at break, and lower elongation at break compared to PBI, as expected considering the slightly higher rigidity, in line with the calorimetric data (PBF has a higher T_g_ than PBI and therefore has lower chain mobility under the experimental conditions). PBI exhibits a T_g_ close to room temperature (22 °C) and is therefore in a rubbery state under the testing conditions, resulting in a greater chain flexibility (Table 3). The possibility of PBF of forming interchain hydrogen bonds could also explain the higher values of the elastic modulus compared to PBI. The elongations at break can be considered high in both cases, owing to the high molecular weight of the materials, indicating good toughness for both polymers.

Regarding the copolymers, in both cases, no appreciable variations in the elastic modulus and stress at break were observed compared to those of the reference homopolymers. Such results are not surprising considering the modest amount of co-units introduced in the two homopolymers (10 mol%), as highlighted by the similar glass transition temperature values of each copolymer and its reference homopolymer (Table 3). The lower rigidity of P(BF_10_BI_90_) compared to P(BF_90_BI_10_) and, in turn, to PBF is consistent with their chemical compositions, with the former containing only a small amount of the more rigid BF moieties.

Interestingly, copolymerization increased the elongation at break, resulting in improved toughness of both copolymers compared to the parent homopolymers. More in detail, the strain at break is higher for P(BF_10_BI_90_) compared to P(BF_90_BI_10_) as a result, once again, of the different chemical composition (higher amount of the more flexible BI moieties) and thermal properties (lower T_g_, i.e., higher chain flexibility).

Finally, for all the materials, the yielding phenomenon can be observed, more remarkably for the polyesters richer in BF co-unit, at an elongation at break of about 2–4% (Figure 3B).

### 2.6. Hydrolytic Degradation Tests

In order to evaluate the stability of the materials under physiological conditions (pH = 7.4, T = 37 °C) for up to 6 months, hydrolytic degradation tests were performed. At the same time, accelerated hydrolytic degradation tests were carried out at 70 °C for up to 60 days, in order to assess the stability under physiological pH (pH = 7.4) for prolonged times. Although no linear correlation exists between degradation times under accelerated and physiological conditions, studies have shown that an incubation time of 60 days at 70 °C corresponds to an incubation time of a few years at 37 °C [57].

From the gravimetric weight loss data, it was found that for both testing conditions, all the materials analyzed did not suffer any loss (less than 1%) at the end of each experiment. This result is particularly encouraging considering the intended application, as the studied materials were designed for long-term permanence inside the human body.

The materials subjected to hydrolytic degradation experiments, together with their relative blank samples, were also characterized thermally (by DSC analysis) to check whether any difference in the main thermal transitions occurred.

The permanence at 37 °C is responsible for the development of a certain amount of crystalline phase, as all the samples analyzed appear to be semicrystalline (Figure S3 and Table S1). In particular, for each material, the DSC profiles of both the blanks (samples incubated at 37 °C but without PBS buffer) and the incubated samples remained almost constant over time. However, some differences between the sets of samples were observed.

In detail, for PBF and P(BF_90_BI_10_), two endothermic phenomena are present: a small multiple one in the temperature range between 55 and 65 °C for the blanks and between 55 and 76 °C for the incubated samples, which is attributable to the presence of a 1D- or a 2D-ordered structure, as reported in the literature for furan-based polyesters [21,26,44]. Moreover, the melting enthalpy was slightly higher for the incubated samples than for their relative blanks, indicating the development of a more perfectly ordered phase due to permanence in water. Conversely, the main endotherm at higher temperatures remained constant for all samples in terms of both position and intensity, suggesting that the main crystalline phase was not altered by incubation in the phosphate buffer.

In both P(BF_10_BI_90_) and PBI, the DSC traces show two endothermic peaks, one at a lower temperature located around 67 °C in the blanks and around 72 °C in the incubated samples. Once again, the permanence in water is responsible for a further increase in crystallinity, as shown by the values of ΔH_m_, which were higher for the incubated samples than for their relative blanks. The principal melting endotherm was centered at 125 °C for P(BF_10_BI_90_) and at 138 °C for PBI, and its intensity did not change over the entire incubation period for both polymers.

As expected, all materials, regardless of the time point considered, appeared semicrystalline after permanence at 70 °C (Figure S4, Table S2). In detail, three endothermic peaks were observed for PBF. The first peak at 56 °C, whose intensity remained constant over time, was always evident in the blank samples; meanwhile, in the samples placed in PBS, it was visible only after 2 days of incubation. The second peak in the blank samples was located at about 96 °C and evolved to 101 °C, while in the incubated samples, it shifted from 107 °C after 2 days to 116 °C after 30 days, and was no longer observable after 60 days. The ΔH_m_ values remained constant. The T_m_ associated with the most intense endothermic phenomena remained in the range of 169–170 °C, and the associated ΔH_m_ values were comparable for the blanks, while they increased in intensity for the incubated samples. This evolution could imply the formation of a more perfect crystalline phase due to the permanence in the phosphate buffer at 70 °C.

For P(BF_90_BI_10_), a similar trend as that observed for PBF is reported, except for the position of the principal melting phenomenon, which remains almost constant in the blank samples (Figure S4, Table S2), while in the hydrolyzed samples, it evolves from 156 °C at day 2 to 165 °C at days 30 and 60.

For P(BF_10_BI_90_) and PBI, only one multiple melting peak was evident, and the higher melting phenomenon remained constant over time. As to the lower-melting peak, for P(BF_10_BI_90_) an evolution from 92 °C to 97 °C in the blank samples and from 98 °C to 105 °C in the incubated ones, respectively, was observed, while for PBI, the smallest peak evolves from 94 °C to 97 °C and from 98 °C to 104 °C for the blank and incubated samples, respectively. This evidence suggests the formation of a more perfect crystalline phase resulting from the permanence at 70 °C.

### 2.7. Surface Properties: Z-Potential and Texture Analysis

**Figure 4:**
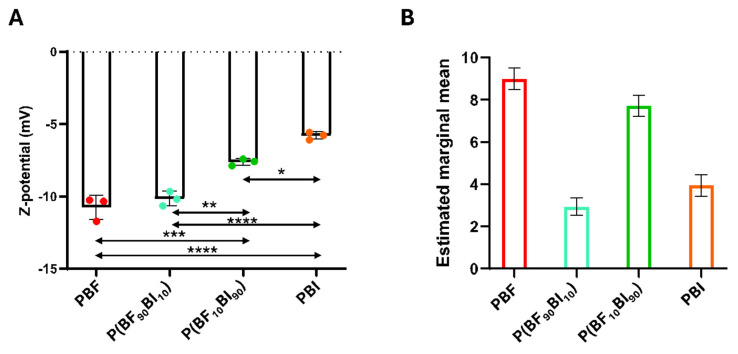
Analysis of surface properties. (**A**) Z-potential measurement at pH 7.4. Data are shown as the mean values of the replicates (*n* = 3) ± the standard deviation (SD), represented by the error bars. One-way analysis of variance (ANOVA), followed by Bonferroni’s test between samples, showed significant difference: *p* value < 0.05 (*); *p* < 0.01 (**); *p* < 0.001 (***) and *p* < 0.0001 (****). (**B**) Haralick’s texture features. Estimated marginal means of PBF, PBI, and P(BF_x_BI_y_) copolymers samples with error bars indicating standard deviation. All comparisons are significant (*p* < 0.05).

The surfaces of the films were also characterized in terms of Z-potential (Figure 4A) and texture analysis (Figure 4B). Zeta potential measurements were conducted to investigate the surface charge properties of these films. The analysis showed that the Z-values were influenced by the chemical composition of the films. Specifically, a significant decrease in the Z value was observed when the BI unit was added (Figure 4A). Indeed, PBF, due to the presence of oxygen within the furan ring, exhibited high polarity, resulting in a more negative Z-potential value. Conversely, PBI displayed a comparatively more positive Z-potential value, due to its non-polar nature [58]. Both copolymers retained properties similar to those of their respective homopolymers; however, the incorporation of the BF co-unit had a more pronounced effect on the copolymer polarity than the addition of the BI unit. A subsequent analysis of the films focused on their surface texture as it appears in SEM images; in particular, the surface texture was evaluated by the estimated marginal means of the 21 Haralick texture features, which obtain information from a gray-level image in terms of pixel contrast and correlation, surface roughness and homogeneity, randomness, and complexity of the surface pattern (Figure 4B). All comparisons were statistically significant (*p* < 0.05). Notably, in a model incorporating covariates (in this work, Haralick’s texture features), an estimated marginal mean constitutes a predicted mean applicable to each case (in this study, the biomaterial type). This predicted mean was derived by calculating the mean of the covariates, thereby ensuring equitable comparisons.

### 2.8. In Vitro Biological Properties

**Figure 5:**
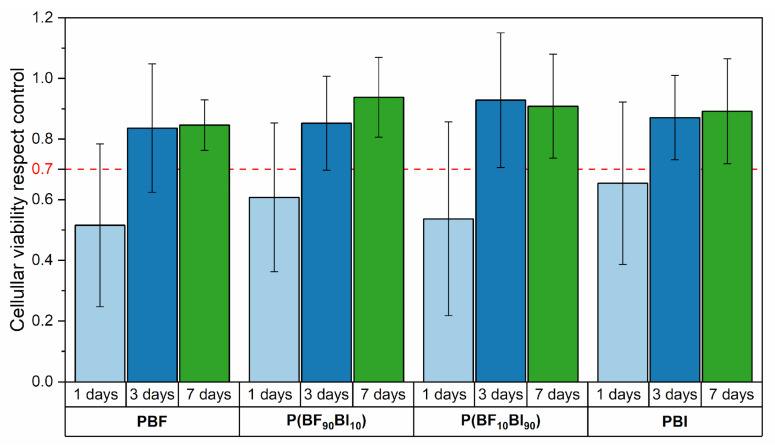
Cell viability of PBF, PBI, and P(BF_x_BI_y_) copolymers, with respect to the control, after 1, 3, and 7 days of incubation. The data are presented as mean value ± SD.

In order to evaluate the applicability of these materials in vascular applications as small blood vessel substitutes, direct-contact in vitro cytotoxicity tests were carried out, using human umbilical vein endothelial cells (HUVECs). Figure 5 shows the cell viability for all materials after 1, 3, and 7 days of incubation, compared to the control (cells cultured in medium without the presence of the polymeric film). As shown, all materials, starting from 3 days after sowing and to an even greater extent after 7 days, showed cell viability above the threshold value of 70%, according to ISO 10993-5 [59], and are therefore non-cytotoxic.

**Figure 6:**
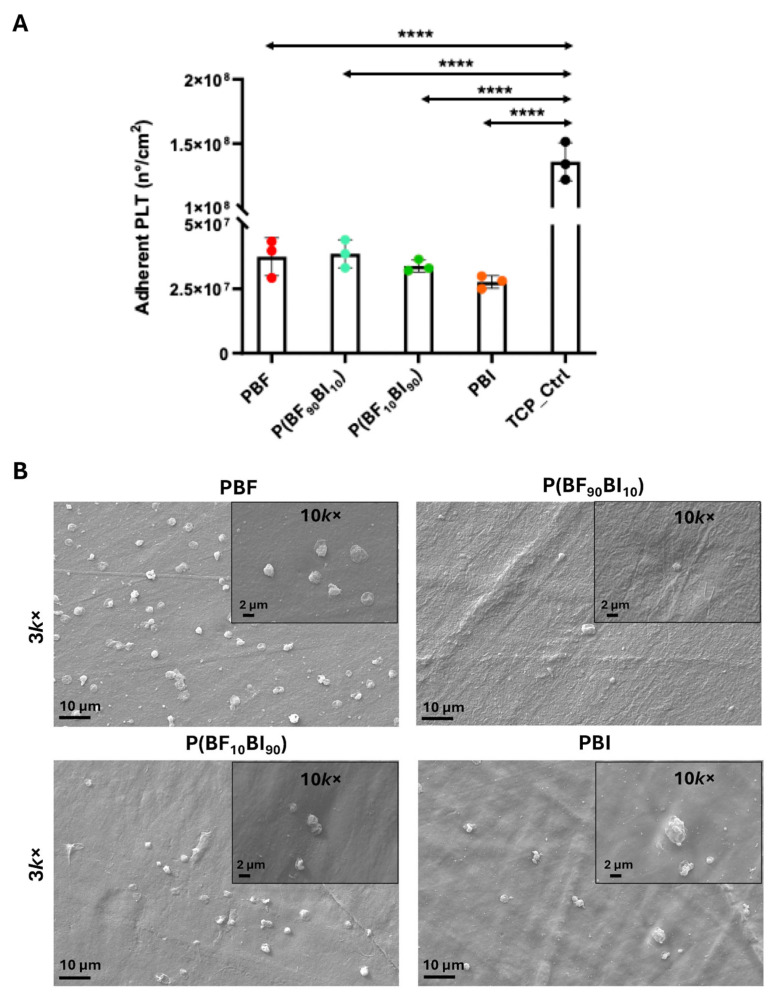
Adhesion of platelets (PLT) on PBF, P(BF_x_BI_y_) copolymers, and PBI. PLT was obtained from Human platelet-rich plasma (hPRP) and incubated for 1 h at 37 °C + 5% CO_2_ on samples and in a tissue culture plate (TCP) well used as a control. (**A**) Adhesion has been determined through the LDH assay. Data were represented as the number of adherent platelets per cm2 of surface. Data are shown as the mean values of the replicates (*n* = 3) ± standard deviation (SD), represented by the error bars. Statistically significant differences of samples vs. TCP were reported: *p* < 0.0001 (****). One-way analysis of variance (ANOVA), followed by Bonferroni’s test between samples, showed no significant differences between surfaces (*p* > 0.05). (**B**) Representative SEM images. Platelets that adhered to the surfaces were fixed and dehydrated to acquire SEM images at 3k× (scale bar 10 µm) magnification and insets at 10k× (scale bar 2 µm). TCP_Ctrl SEM images were in Supplementary Figure S5.

In addition, the biological properties of the surfaces included the evaluation of PBF, PBI, and P(BF_x_BI_y_) surfaces in terms of their interaction with platelets. Platelet adhesion, activation and aggregation are involved in blood coagulation, particularly in clot formation [60], and are particularly relevant for the design of engineered surfaces proposed as biomedical devices, such as blood catheters [61]. Figure 6A shows the data on platelet adhesion on the surfaces, represented as the number of adherent platelets per cm^2^ of surface. The LDH assay revealed a decreasing trend in platelet adhesion from PBF to PBI, although no significant differences were observed between the tested materials (*p* > 0.05). Significant differences were found between the TCP control and the samples (* *p* < 0.05). Interestingly, a comparison of the number of platelets adhering to the surfaces and platelet seeded (per cm^2^) showed that approximately 6% of the platelets adhered to the PBF and P(BF_90_BI_10_) surfaces, and around 4%, 5%, and 20% to the P(BF_10_BI_90_), PBI surfaces, and TCP control, respectively. Qualitative SEM images (Figure 6B) of platelet adhesion on surfaces confirmed the quantitative data: few platelets were observed on all surfaces and in smaller amounts than those of the TCP control (Figure S5), and in all samples, attached platelets did not show pseudopodia formation, a known sign of platelet activation [62,63,64]. Chemistry, ionic charge, topography, and surface wettability of materials can be used as effective surface modifiers to improve hemocompatibility by promoting the downregulation of platelet adhesion [65,66]. In agreement with the literature, which reports that materials with hydrophobic properties have better blood compatibility and lower platelet adhesion and activation potential [40,67,68,69,70], the low platelet adhesion found here can be explained by the hydrophobic nature of the synthesized materials. Similarly, the highest hydrophobicity value of PBI may explain the trend of decreased platelet adhesion from PBF to PBI.

**Figure 7:**
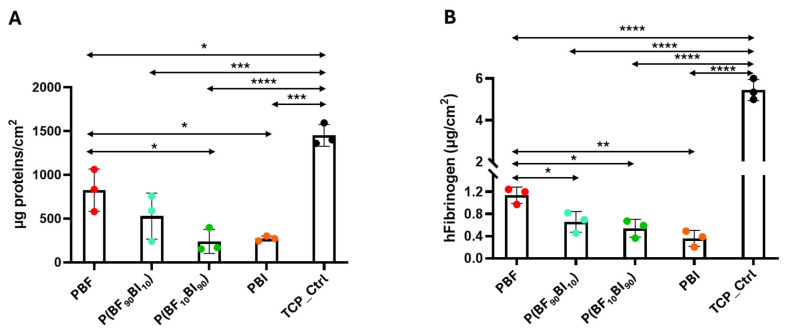
Human plasma total protein and fibrinogen absorption. Human plasma was immobilized on samples and on a tissue culture plate (TCP) control for 1 h at 37 °C. After the incubation time, the plasma proteins absorbed from the material’s surface were quantified (**A**) through the colorimetric BCA assay and expressed as µg proteins/cm^2^. The quantity of human plasma fibrinogen absorbed from the surfaces (**B**) was detected through ELISA assay and expressed as µg/cm^2^. Error bars indicate the standard deviation (SD) of the mean values of the replicates (*n* = 3). One-way analysis of variance (ANOVA) (*), followed by Bonferroni’s test between samples (*p* value < 0.05), was performed: *p* value < 0.05 (*); *p* < 0.01 (**); *p* < 0.001 (***) and *p* < 0.0001 (****). In (**A**) PBF vs. P(BF_90_BI_10_) samples: *p* value > 0.05; P(BF_90_BI_10_) vs. P(BF_10_BI_90_): *p* value > 0.05; PBI vs. P(BF_x_BI_y_): *p* value > 0.05. In (**B**) P(BF_90_BI_10_) vs. P(BF_10_BI_90_): *p* value > 0.05; PBI vs. P(BF_x_BI_y_): *p* value > 0.05.

Protein adsorption is the first significant event following blood contact with ‘foreign’ surfaces and is considered a precursor to subsequent events, such as platelet adhesion, activation, aggregation, plasma coagulation, immune responses, and inflammation [71]. Unfortunately, these effects may represent the most serious limitation of blood-contact devices [72]. Fibrinogen, a major coagulation factor in plasma, has been recognized as one of the most important types of adsorbed proteins involved in platelet binding and activation, and its adsorption can be used as a measure of material hemocompatibility [73]. To assess the ability to bind plasma proteins, samples were incubated with human plasma to quantify the amount of total proteins and fibrinogen absorbed using the BCA protein assay (Figure 7A) and ELISA (Figure 7B), respectively. In terms of total protein absorption, significant differences were observed between PBF and P(BF_10_BI_90_) (* *p* < 0.05) and PBI (* *p* < 0.05), whereas no difference was found between PBF and P(BF_90_BI_10_) (*p* > 0.05) (Figure 7A). The PBF surface exhibited the most pronounced protein adsorption capacity (around 700 µg/cm^2^) compared to the other films (all < 500 µg/cm^2^) (Figure 7A). However, it is important to note that all these values are less than 1 per cent of the total plasma protein concentration (60–90 mg/mL), which suggests that, although they were analyzed under static conditions (not conditions mimicking blood flow), these surfaces can absorb a reduced amount of total plasma protein. Regarding fibrinogen absorption, a significantly higher amount of fibrinogen absorption was determined in PBF compared to both copolymers (* *p* < 0.05) and PBI (** *p* < 0.01) (Figure 7B). A very relevant finding is that the amount of fibrinogen quantified in all materials (less than 1 µ/cm^2^) was lower than the values determined for FDA-approved materials for contact with blood (e.g., poly(tetrafluoroethylene), around 90 µg/cm^2^) [74]. In addition, two other observations are important to note: both plasma protein and fibrinogen adsorption values were lower than those in the control (*p* < 0.05), and a trend toward decreased adsorption was observed with the addition of the BI unit. This implies that both phenomena were influenced by the nature of the surface. This is not surprising since the scientific literature demonstrates that surface chemistry, hydrophobicity, and hydrophilicity drastically influence the amount/conformation of the adsorbed fibrinogen molecule, although whether positively or negatively is not yet very clear given the non-coherent findings [65,70,71]. In this study, we hypothesize that the decrease in polarity from the furan ring (PBF) to the isophthalic ring (PBI) is potentially responsible for counteracting the absorption of both plasma proteins and fibrinogen, and thus is crucial in preventing the adhesion of platelets. However, it cannot be ruled out that the surface charge also plays a role in this process. Surface charge is another property of materials currently being studied, as it has been shown in other studies how it can play a role in both the absorption process of plasma proteins, such as fibrinogen, and platelet adhesion [75,76]. Although further studies are needed, the data are particularly significant because they reflect researchers’ enormous efforts to find a technical solution to improve surface properties to prevent platelet adhesion and subsequent activation by implantable devices [77].

Finally, the amount of absorbed plasma fibrinogen was correlated with Haralick’s texture analysis. The correlation coefficient between the estimated marginal averages and fibrinogen concentrations on the surface of the biomaterials was approximately 0.73. This positive correlation indicates that as the estimated marginal mean decreases, the adsorption of fibrinogen on the biomaterial surface also decreases.

## 3. Materials and Methods

### 3.1. Materials

1,4-butanediol (BD, Sigma Aldrich, Saint Louis, MO, USA), dimethyl 2,5-furandicarboxylate (DMF, Sarchem Labs, Farmingdale, NJ, USA), dimethyl isophthalate (DMI, Sigma Aldrich, Saint Louis, MO, USA), titanium tetrabutoxide (TBT, Sigma Aldrich, Saint Louis, MO, USA), and titanium isopropoxide (TIP, Sigma Aldrich, Saint Louis, MO, USA) were all used as purchased.

### 3.2. Synthesis

The homopolymers poly(butylene 2,5-furanoate) (PBF) and poly(butylene isophthalate) (PBI) were synthesized by two-stage bulk polycondensation from 1,4-butanediol (BD) and dimethyl 2,5-furandicarboxylate (DMF), in the case of PBF, and dimethyl isophthalate (DMI) in the case of PBI, in the presence of the catalysts TBT and TIP (400 ppm/g polymer each) and a 20 mol% glycolic excess. In the case of the P(BF_x_BI_y_) copolymers, the reagents used were the same as those above, but the relative molar amounts of the two diesters varied depending on the copolymer, maintaining a 20 mol% excess of glycol. The reagents and catalyst were charged in a continuously stirred glass reactor and placed in a thermostatic bath. The first step (transesterification stage) was carried out at 195 °C under a flow of pure nitrogen, while methanol was distilled off. The second step (polycondensation stage) was carried out under vacuum to increase the molecular weight of the final polymer. In this last stage, the temperature was gradually increased from 195 to 220 °C, while the pressure was slowly decreased to 0.05 mbar, and a progressive increase in the torque value was observed. The synthesis was stopped once a constant value of the measured torque was recorded and no distillation was observed.

Prior to further characterization and processing, the obtained polymers were purified by dissolution in chloroform (adding a few drops of 1,1,1,3,3,3-hexafluoro-2-propanol to allow complete solubilization of the materials, when needed) and further precipitation in a large molar excess of methanol under stirring.

### 3.3. Molecular Characterization

The chemical structure and composition were determined using ^1^H-NMR. The samples were dissolved at a concentration of 10 mg/mL in deuterated chloroform with tetramethylsilane (TMS, 0.03 vol%) as the internal standard. The measurements were carried out at 25 °C using a Varian INOVA 400 MHz instrument (Palo Alto, CA, USA).

Gel permeation chromatography (GPC) was used to evaluate the molecular weight (M_n_) and polydispersity index (PDI) of the synthesized polymers. The measurements were performed at 30 °C using an HPLC Lab Flow 2000 apparatus (Waters, Milford, MA, USA) equipped with a Rheodyne 7725i injector (Thermo Fisher Scientific, Waltham, MA, USA), Phenogel MXM 5 μm mixed-bed column (Torrence, CA, USA), and KNAUER RI K-2301 (Berlin, Germany) detector.The instrument was calibrated with polystyrene standards in the range of 550–2,500,000 g/mol, and HPLC-grade chloroform was used as the eluent (flow of 1 mL/min, concentration of the samples of 2 mg/mL).

The intrinsic viscosity (η) was determined using a Ubbelohde viscometer 31 13/Ic (diameter 0.84 mm) at 30 °C. Flow-time measurements were conducted using an electronic sensor. Four different concentrations of polymer in phenol/1,1,2,2-tetrachloroethane (60/40% *w*/*w*) solutions (0.8, 0.7, 0.6, and 0.5 g/dl) were used, and the measurements were repeated at least five times. The intrinsic viscosity (η) was obtained by extrapolating the linear plots of (ln η_r_)/C and η_sp_/C, where η_r_ and η_sp_ are the relative and specific viscosities, respectively, and C is the concentration.

### 3.4. Film Preparation

All purified samples were compression-molded into films using a Carver (Wabash, IN, USA) C12 lab press. The polymers were placed between two Teflon plates, heated to a temperature 10 °C higher than their melting temperature, and allowed to melt for a couple of minutes. Afterwards, a pressure of 5 ton/m^2^ was applied and maintained for 1 min, and the films were ballistically cooled to room temperature under pressure.

Prior to further characterization, the films were stored at room temperature for two weeks in order to allow them to reach crystallization equilibrium.

### 3.5. Thermal Analysis

Thermal stability was evaluated using thermogravimetric analysis (TGA) with a PerkinElmer TGA4000 instrument (Waltham, MA, USA). The measurements were carried out under a nitrogen atmosphere (40 mL/min) by heating weighed samples of about 5 mg from 40 to 800 °C at a rate of 10 °C/min. The temperature of the maximum degradation rate, T_max_, was calculated from the TGA derivatives as the minimum of the peak, while the temperature of initial degradation, T_id_, represents the temperature at which degradation starts.

To define the characteristic thermal transitions of both the purified powders and films, differential scanning calorimetry (DSC) was carried out under a nitrogen atmosphere (20 mL/min) using a PerkinElmer DSC6 instrument (Waltham, MA, USA) equipped with an intracooler able to reach −70 °C. Samples of 8 mg were first heated from −50 °C to T_m_ + 20 °C at 20 °C/min (I scan), held for 3 min, and then rapidly cooled to −50 °C at 100 °C/min; afterwards, a second heating step was applied from −50 °C to T_m_ + 20 °C at 20 °C/min (II scan).

The Glass transition temperature (T_g_) was determined at half-height of the glass-to-rubber transition step, while the corresponding heat associated (ΔC_p_) was calculated from the step height. The melting temperature (T_m_) was taken as the maximum of the endothermic melting peak, and the associated enthalpy (ΔH_m_) was calculated from the peak area. Similarly, the cold crystallization temperature (T_cc_) and the corresponding enthalpy (ΔH_cc_) were evaluated.

### 3.6. Wide Angle X-Ray Scattering (WAXS)

Wide-angle X-ray scattering (WAXS) analysis was performed on the purified powders at room temperature using a PANalytical X’Pert PRO diffractometer (Almelo, The Netherlands) equipped with an XCelerator detector and a Cu anode as the X-ray source (λ_1_ = 0.15406 nm, λ_2_ = 0.15443 nm). The degree of crystallinity (χ_c_) was evaluated as the ratio of the areas of the crystalline peaks to the total diffraction area under the scattering curve using the HighScore software 4.9 release (PANalytical, Almelo, The Netherlands). The crystal size was evaluated from the width of the peaks at 18.2° (PBF) and 16.8° (PBI) using the Scherrer equation.

### 3.7. Water Contact Angle (WCA) Measurements

Static water contact angle (WCA) measurements were performed on flat film surfaces using a Krüss (Hamburg, Germany) DSA30S instrument equipped with Drop Shape Analysis software. Each sample was washed in a 70% *v*/*v* ethanol solution and dried overnight at room temperature. The profile images of deionized water drops (4 μL, 100 μL/min) were acquired after 1 s from deposition, and the WCA values were measured using the software. At least 10 drops were analyzed for each film, and the WCA values were provided as the mean value ± standard deviation.

### 3.8. Mechanical Characterization

Tensile testing was performed using an Instron 5966 machine (Norwood, MA, USA) equipped with rubber grips and a 10 kN load cell controlled by a computer. Rectangular stripes (5 × 50 mm, gauge length of 20 mm) were employed for the tests, and a crosshead speed of 10 mm/min was adopted.

For the tensile testing, the stress at break (σ_B_) and elongation at break (ε_B_) were directly determined from the end points of the relative stress-strain curves, while the tensile elastic modulus (E) was calculated from the slope of the initial linear part. The stress at yield (σ_Y_) and elongation at yield (ε_Y_) were determined by evaluating the meeting point between the stress and strain curve and a straight line with a modulus equal to the calculated elastic modulus, horizontally shifted by 1% with respect to the linear section of the stress-strain curve.

For the tests, at least six specimens were analyzed, and the results were provided as the mean value ± standard deviation.

### 3.9. Hydrolytic Degradation Tests

Hydrolytic degradation tests were carried out in phosphate buffered saline (PBS, 137 mM NaCl, 2.7 mM KCl, 4.3 mM Na_2_HPO_4_, 1.4 mM KH_2_PO_4_, pH 7.4) at 70 °C (accelerated condition) for 60 days and at 37 °C (physiological conditions) for 6 months, according to ISO 10993-13 [78]. For each material under examination, rectangular samples (0.5 × 2 mm) were obtained and weighed, then immersed in 2.5 mL of PBS and incubated at 70 °C in an MPM Instruments (Bernareggio (MB), Italy) M-80 VF oven and at 37 °C in a Stuart (Cole-Parmer Ltd., Staffordshire, UK) SI500 incubator, respectively. Blank samples were also incubated at the same temperatures but without PBS for comparison. The buffer solution was periodically changed to maintain a constant pH throughout the experiment. Gravimetric weight-loss measurements and DSC analyses of the withdrawn samples were also performed.

### 3.10. Surface Zeta Potential

The zeta potential (ζ) of the samples was determined using a SurPASS™ 3 (Anton Paar GmbH, Graz, Austria). The streaming potential was measured at physiological pH, as previously reported [79]. The samples (75 mm^2^) were placed between two filter disks in the sample holder of a cylindrical cell. An aqueous solution of 0.01 M potassium chloride was used as the streaming solvent, and ζ was measured at pH 7.4.

### 3.11. Texture Features

Scanning electron microscopy images of the biomaterials were acquired using a Zeiss EVO-MA10 scanning electron microscope (Zeiss, Oberkochen, Germany) with an accelerating voltage of 20 kV and 5k× magnification to analyze the texture features of the biomaterial surfaces. The analysis was evaluated via a Computer Vision approach, in particular by the so-called Haralick’s texture features [80], implemented with new formulas given by Löfstedt et al. [81] as reported in [82]. From the SEM images, 21 Haralick’s texture features were extracted using a custom-made script written in the Matlab^®^ Programming Language (Release R2024b, The MathWorks, Inc., Natick, MA, USA). Multivariate repeated measures statistics based on Tukey’s Honest Significant Difference procedure were also performed. Each one-to-one comparison between biomaterials was expressed in terms of their estimated marginal means and *p*-values (significance level of 0.05).

### 3.12. In Vitro Biological Evaluation

#### 3.12.1. Cytotoxicity Tests

Human umbilical vein endothelial cells (HUVECs) were routinely cultured in Dulbecco’s Modified Eagle Medium (DMEM) supplemented with 10% fetal bovine serum (FBS) in an incubator at 37 °C and 5% CO_2_. Before the experiment, the polymeric films (1 × 1 cm) were sterilized in aqueous solutions containing increasing concentrations of ethanol (70 and 90 vol%) for 30 min each and then washed twice with PBS and cell culture medium for 10 min.

HUVECs were seeded in a 24-well plate at a density of 5000/cm^2^ for each well, and after 5 h of incubation, the sterilized polymers were placed inside the wells. The plate was then incubated at 37 °C and 5% CO_2_. The viability of HUVECs was evaluated using a 3-[4,5-dimethylthiazol-2-yl]-2,5-diphenyl tetrazolium bromide (MTT) assay, which was carried out after 1, 3, and 7 days of incubation. Briefly, at the desired time points, both the polymer and cellular medium were removed from the wells, 450 µL of DMEM and 50 μL of MTT solution were added to the cells, and after 2.5 h, 500 µL of lysis buffer (0.1 g/mL sodium dodecyl sulfate, 41.67 µL/50 mL HCl 0.01 M) was added. The following day, 200 μL aliquots of the solution were sampled. Absorbance values were measured using a microplate reader at 570 nm.

#### 3.12.2. Platelet Adhesion

Human platelet-rich plasma (hPRP) derived from healthy donors was obtained from the Fondazione IRCCS Policlinico San Matteo, Pavia (Italy). hPRP was isolated according to “Decreto Ministero della Salute 2 November 2015 n.69, Disposizioni relative ai requisiti di qualità e sicurezza del sangue e degli emocomponenti” and “Accordo Stato-Regioni n.225/CSR 13 December 2018, Schema-tipo di convenzione per la cessione del sangue e dei suoi prodotti per uso di laboratorio e per la produzione di dispositivi medico-diagnostici in vitro”. The experiment was performed as described by Restivo et al. [82]. Briefly, human platelets (2 × 10^8^ platelets/mL) were seeded on sterile PBF, PBI, and P(BF_x_BI_y_) samples for 1 h at 37 °C. After incubation, the samples were treated as previously reported [82], and the platelets were quantified using a lactate dehydrogenase (LDH) assay (Sigma-Aldrich, St. Louis, MO, USA). The experiment was performed in triplicate. A titration curve with a known concentration of platelets/mL was used to plot the obtained absorbance. For qualitative evaluation, scanning electron microscopy (SEM) images were acquired of the platelets adhering to the surfaces. Samples were incubated with hPRP for 1 h, gently washed with PBS 1X, fixed with glutaraldehyde 2.5% (*v*/*v*) in Na-cacodylate buffer, and washed and dehydrated with 25 %, 50%, 75%, and 96% ethanol [83]. Platelets seeded on plastic cell culture coverslip disks (Thermanox Plastic, Nalge Nunc International, Rochester, NY, USA) were used as the control. SEM images were acquired at 3k× (scale bar = 10 µm) and 10k× (scale bar = 2 µm) magnifications.

#### 3.12.3. Protein Quantification

##### Human Plasma Proteins Absorption

Human plasma derived from healthy donors was immobilized on PBF, PBI, and P(BF_x_BI_y_) samples. Briefly, 100 μL of plasma solution was incubated with the samples for 1 h at 37 °C. After incubation, the samples were gently washed, and the immobilized plasma proteins were quantified using a bicinchoninic acid (BCA) protein assay kit (Pierce Biotechnology, Inc., Rockford, USA), following the manufacturer’s instructions. The experiment was performed in triplicate, and a sample of each type was used as a negative control. Human plasma immobilized on a high-binding 96-well plate was used as a positive control and indicated as TCP (tissue culture plate). The obtained absorbance was related to a calibration curve containing known amounts of a standard protein and expressed as μg/cm^2^.

##### Human Fibrinogen Absorption

Human plasma fibrinogen (hFbg) adsorption assay was performed using an Enzyme-linked Immunosorbent assay (ELISA). Briefly, 100 μL of plasma solution from healthy adult donors was incubated as previously described for samples and in TCP wells used as a control. BSA-coated wells were used as the negative controls. After incubation, each sample was washed and treated as described by [82]. The reaction was developed with 200 μL/well of OPD tablets (Sigma-Aldrich, St. Louis, MO, USA) and dissolved in double-distilled water. The absorbance was measured at 450 nm with 620 nm as the reference wavelength using a CLARIOstar^®^ Plus Multi-mode Microplate Reader (BMG Labtech, Ortenberg, Germany). The experiment was performed in triplicate. A calibration curve was constructed by gradually increasing the concentrations of purified human fibrinogen protein (from 500 ng to 10 μg/mL) to determine the amount of hFbg in each sample. The results were expressed as [μg/mL]/cm^2^ [82]. In addition, the plasma fibrinogen concentrations [μg/cm^2^] absorbed by each biomaterial surface were mathematically correlated to the estimated marginal means derived from the texture analysis (Section 3.11).

### 3.13. Statistical Analysis

Statistical analysis of the Z-potential measurements and the experiments conducted with human plasma was performed by calculating the mean of the results (in triplicate) obtained from two independent experiments. The analysis was conducted using GraphPad Prism 9 software (GraphPad Inc., Boston, MA, USA). A one-way analysis of variance (ANOVA), followed by Bonferroni’s multiple comparison test, was performed. *p* values *<* 0.05 were considered statistically significant [84].

## 4. Conclusions

In this study, a family of two aromatic homopolymers and two random copolymers, namely PBF, PBI, and P(BF_x_BI_y_) where x and y are the relative molar amounts of the two co-units, have been successfully synthesized by two-step melt polycondensation, and investigated for their possible use in the treatment of vessels with a diameter smaller than 6 mm. The starting homopolymers were properly chosen, as characterized by high chemical stability, a necessary requirement for long-term applications. In order to tune the chemical-physical and functional properties of these two homopolymers, copolymerization was carried out. In detail, through the insertion of only 10 mol% of co-units, it was possible to improve the toughness of both PBF and PBI while maintaining at the same time their high thermal stability, which was comparable to that of other polymers used for the fabrication of vascular grafts. Stability over time under both physiological and accelerated conditions was confirmed in all cases.

For applications inside the human body, proper surface characteristics and biocompatibility should be fulfilled. Accordingly, no cytotoxicity was observed, even after 7 days of incubation, while a modulation of the surface properties was measured as a function of the chemical composition. Finally, since blood contact is expected for these materials, preliminary hemocompatibility tests were also performed, showing very low platelet adhesion, as well as lower protein and fibrinogen absorption for PBF compared to the control. The above-mentioned phenomena further decreased as the amount of BI co-unit increased. This noteworthy behavior can be ascribed to the synergistic effect of both the peculiar surface chemistry and the hydrophobic character of these materials, according to the WCA and Z-potential measurements.

Although further studies are needed, the results obtained to date are meaningful and allow us to conclude that the proper chemical design was effective in tuning the properties of the reference homopolymers in order to obtain materials with suitable features for applications in vascular tissue engineering.

## Figures and Tables

**Table 1:** Molecular characterization data (^1^H–NMR, I.V., and GPC) of PBF, PBI, and P(BF_x_BI_y_) copolymers.

Sample	^1^H-NMR		Viscosimetry	GPC	
	BF Feed Mol%	BF Actual Mol%	I.V. dL/g	M_n_ g/mol	PDI -
PBF	100	100	1.13	/	/
P(BF_90_BI_10_)	90	91	0.97	/	/
P(BF_10_BI_90_)	10	10	0.77	50,000	1.7
PBI	/	/	0.89	55,000	1.6

**Table 2:** Thermal (TGA and DSC) and structural (WAXS) characterization data of PBF, PBI, and P(BF_x_BI_y_) copolymers in the form of purified powders.

Sample	TGA		DSC								WAXS	
			I SCAN		II SCAN							
	T_id_	T_max_	T_m_	ΔH_m_	T_g_	ΔC_p_	T_cc_	ΔH_cc_	T_m_	ΔH_m_	X_c_	C.S.
	°C	°C	°C	J/g	°C	J/g °C	°C	J/g	°C	J/g	%	nm
PBF	378	392	170	40	34	0.364	95	34	169	40	36	5
P(BF_90_BI_10_)	375	392	156	36	33	0.432	110	28	155	28	29	4
P(BF_10_BI_90_)	387	409	125	31	24	0.354	/	/	/	/	34	10
PBI	389	409	142	38	22	0.381	/	/	/	/	37	10

**Table 3:** Thermal (DSC) and surface wettability (WCA) characterization data of PBF, PBI, and P(BF_x_BI_y_) copolymers in the form of compression-moulded films.

Sample	DSC—I Scan						WCA
	T_g_	ΔC_p_	T_cc_	ΔH_cc_	T_m_	ΔH_m_	°
	°C	J/g °C	°C	J/g	°C	J/g	
PBF	34	0.352	92	40	170	40	92 ± 3
P(BF_90_BI_10_)	33	0.245	102	34	154	34	92 ± 3
P(BF_10_BI_90_)	24	0.437	/	/	/	/	91 ± 3
PBI	22	0.367	/	/	/	/	96 ± 2

**Table 4:** Mechanical characterization data of PBF, PBI, and P(BF_x_BI_y_) copolymers.

Sample	E	σ_Y_	ε_Y_	σ_B_	ε_B_
	MPa	MPa	%	MPa	%
PBF	1186 ± 99	26 ± 3	4.1 ± 0.2	28 ± 4	290 ± 99
P(BF_90_BI_10_)	1223 ± 127	34 ± 4	4.1 ± 0.4	32 ± 6	498 ± 99
P(BF_10_BI_90_)	449 ± 13	5.9 ± 0.2	2.4 ± 0.2	25 ± 6	926 ± 98
PBI	426 ± 20	4.3 ± 0.5	3.9 ± 0.8	23 ± 4	795 ± 38

## Data Availability

Raw data are available from the corresponding authors upon reasonable request.

## Supplementary Materials

The following supporting information can be downloaded at: https://www.mdpi.com/article/10.3390/ijms26136470/s1.
