# Supplementary material for: Design and Characterization of Aromatic Copolyesters Containing Furan and Isophthalic Rings with Suitable Properties for Vascular Tissue Engineering
Source: Int J Mol Sci. 2025 Jul 4;26(13):6470. doi: 10.3390/ijms26136470 (PMC12249642; doi:10.3390/ijms26136470)
Supplement: Supplementary file 1 [file ijms-26-06470-s001.zip › ijms-3694146-supplementary.pdf]

SUPPLEMENTARY INFORMATION

Design and Characterization of Aromatic Copolyesters Containing Furan and Isophthalic Rings with Suitable Properties for Vascular Tissue Engineering

Edoardo Bondi [1], Elisa Restivo [2], Michelina Soccio [1], Giulia Guidotti [1,*], Nora Bloise [2,3,*], Ilenia Motta [4], Massimo Gazzano [5], Marco Ruggeri [6], Lorenzo Fassina [7], Livia Visai [2,3], Gianandrea Pasquinelli [4,8] and Nadia Lotti [1]

[1] Department of Civil, Chemical, Environmental, and Materials Engineering, University of Bologna,
Via Terracini 28, 40131 Bologna, Italy; edoardo.bondi3@unibo.it (E.B.); m.soccio@unibo.it (M.S.); nadia.lotti@unibo.it (N.L.)

[2] Molecular Medicine Department (DMM), Centre for Health Technologies (CHT), Unità di Ricerca (UdR) INSTM, Operative Unit (OU) of Interuniversity Center for the Promotion of the 3Rs Principles in Teaching and Research (Centro 3R), University of Pavia, 27100 Pavia, Italy; elisa.restivo01@universitadipavia.it (E.R.); livia.visai@unipv.it (L.V.)

[3] UOR6 Nanotechnology Laboratory, Department of Prevention and Rehabilitation in Occupational Medicine and Specialty Medicine, Istituti Clinici Scientifici Maugeri IRCCS, 27100 Pavia, Italy

[4] Department of Medical and Surgical Sciences (DIMEC), University of Bologna, Via Massarenti 9, 40138 Bologna, Italy; ilenia.motta2@unibo.it (I.M.); gianandr.pasquinelli@unibo.it (G.P.)

[5] Institute for Organic Synthesis and Photoreactivity, ISOF-CNR, Via Gobetti 101, 40129 Bologna, Italy;
massimo.gazzano@isof.cnr.it

[6] Department of Drug Sciences, University of Pavia, Viale Taramelli 12, 27100 Pavia, Italy; marco.ruggeri@unipv.it

[7] Department of Electrical, Computer and Biomedical Engineering, University of Pavia, Via Ferrata 5,
27100 Pavia, Italy; lorenzo.fassina@unipv.it

[8] Pathology Unit, IRCCS Azienda Ospedaliero-Universitaria di Bologna, 40138 Bologna, Italy

* Correspondence: giulia.guidotti9@unibo.it (G.G.); nora.bloise@unipv.it (N.B.)

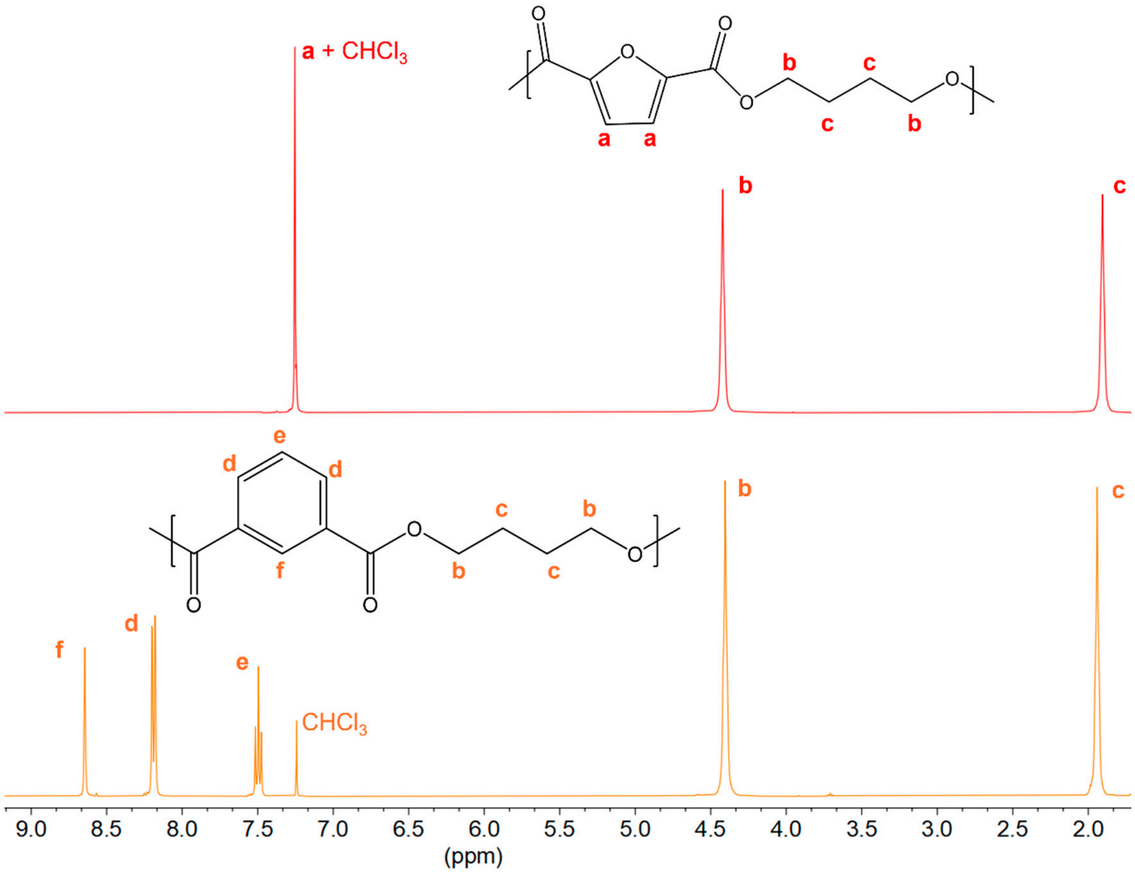

Figure S1. ^{1}H-NMR spectra of PBF and PBI with peak assignments.

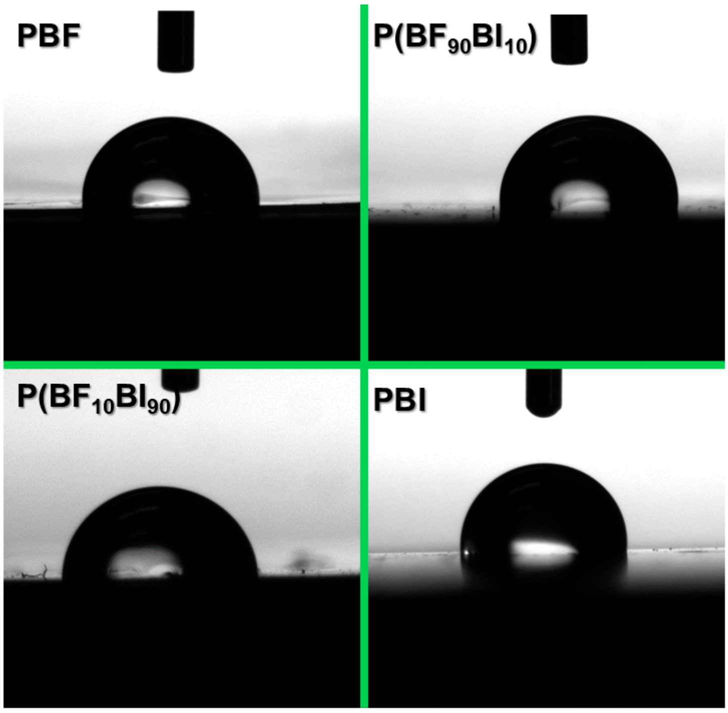

Figure S2. Pictures of water drops on PBF, PBI, and P(BF_xBI_y) films surfaces

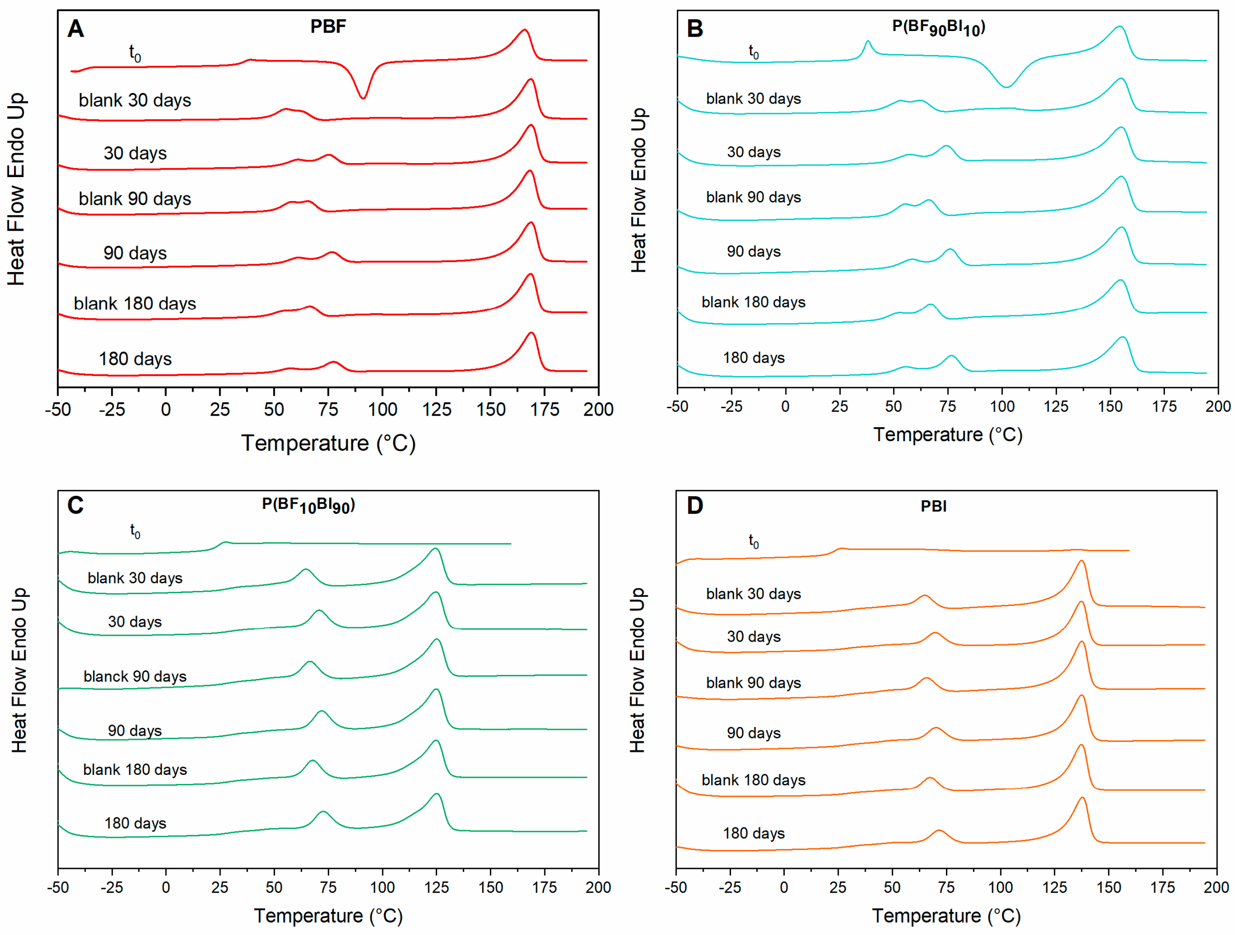

Figure S3.A-D) I DSC scans of PBF, PBI, and P(BF$_x$BI$_y$) copolymeric films before and after hydrolytic tests at 37°C (after 30, 90 and 180 days of incubation), together with those of the relative blanks

Table S1. Thermal characterization data (DSC) of PBF, PBI, and P(BF_xBI_y) copolymeric films before and after hydrolytic tests at 37 °C (after 30, 90 and 180 days of incubation), together with those of the relative blanks

	Days	T_{m1} °C	T_{m2} °C	ΔH_{m1+2} J/g	T_{m3} °C	ΔH_{m3} J/g
**PBF**						
	30 blank	55	61	12	168	42
	30	55	75	15	168	40
	90 blank	61	65	15	168	40
	90	55	77	16	169	42
	180 blank	58	66	14	168	41
	180	61	77	15	169	41
**P($BF_{90}BI_{10}$)**						
	30 blank	53	62	12	155	30
	30	57	74	17	155	31
	90 blank	55	66	16	155	31
	90	58	76	17	155	33
	180 blank	52	67	14	155	30
	180	55	76	16	156	31
**P($BF_{10}BI_{90}$)**						
	30 blank		65	8	125	26
	30		71	9	125	27
	90 blank		67	8	125	26
	90		72	10	125	27
	180 blank		68	8	125	26
	180		73	9	125	26
**PBI**						
	30 blank		65	6	137	31
	30		70	7	137	30
	90 blank		66	7	138	32
	90		70	7	138	31
	180 blank		67	8	137	30
	180		72	8	138	31

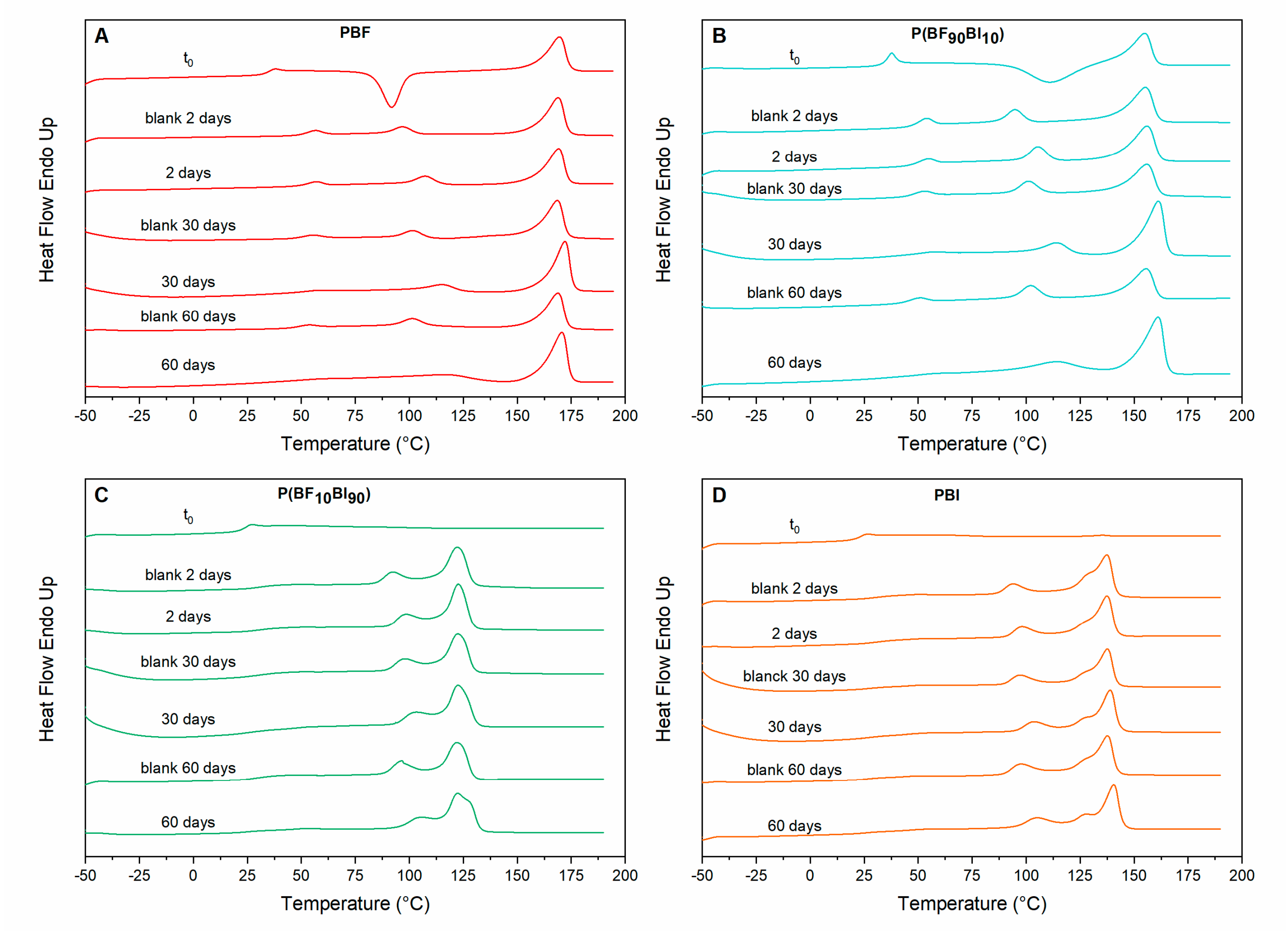

Figure S4. A-D) I DSC scans of PBF, PBI, and P(BF_xBI_y) copolymeric films before and after hydrolytic tests at 70 °C, together with those of the relative blanks

Table S2. Thermal characterization data (DSC) of PBF, PBI, and $P(BF_xBI_y)$ copolymeric films before and after hydrolytic tests at 70 °C, together with those of the relative blanks

	Days	T_{m1} °C	ΔH_{m1} J/g	T_{m2} °C	ΔH_{m2} J/g	T_{m3} °C	T_{m4} °C	ΔH_{m3+4} J/g
**PBF**								
	2 blank	56	3	97	6		169	40
	2	57	2	107	7		169	38
	30 blank	55	2	102	6		169	39
	30			116	6		172	49
	60 blank	55	2	102	6		169	39
	60						171	52
**$P(BF_{90}BI_{10})$**								
	2 blank	54	3	95	8		155	28
	2	54	3	105	9		156	29
	30 blank	52	2	101	7		156	26
	30			114	8		161	42
	60 blank	51	2	102	8		156	23
	60			115	8		161	44
**$P(BF_{10}BI_{90})$**								
	2 blank					92	122	46
	2					98	123	45
	30 blank					98	122	43
	30					102	122	46
	60 blank					97	122	41
	60					105	122	46
**PBI**								
	2 blank					94	137	49
	2					98	137	45
	30 blank					97	138	41
	30					104	139	44
	60 blank					97	138	46
	60					105	141	47

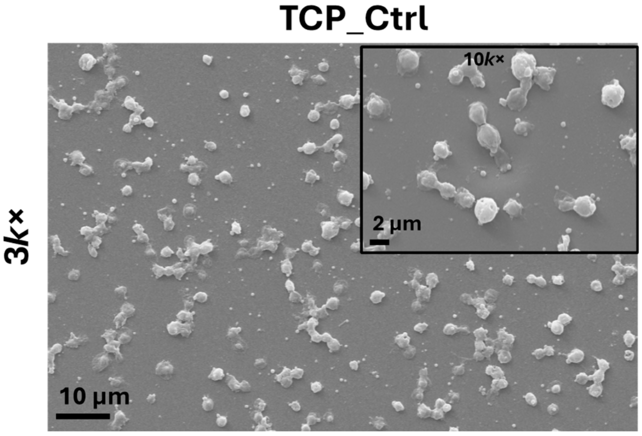

Figure S5. Representative *SEM image of platelet adhesion (PLT) on a tissue culture plate (TCP) well used as a control.* Platelets adhered were fixed and dehydrated to acquired SEM images at 3k× (scale bar 10 μm) magnification and insets at 10k× (scale bar 2 μm)
